# Explaining the role of the social determinants of health on health inequality in South Africa

**DOI:** 10.3402/gha.v8.28865

**Published:** 2015-09-16

**Authors:** John Ele-Ojo Ataguba, Candy Day, Di McIntyre

**Affiliations:** 1 Health Economics Unit, School of Public Health and Family Medicine, University of Cape Town, Cape Town, South Africa; 2 Health Systems Trust, Durban, South Africa

**Keywords:** social determinants of health, self-assessed health, health inequality, inequality decomposition, inter-sectoral action, South Africa

## Abstract

**Background:**

Action on the social determinants of health (SDH) is relevant for reducing health inequalities. This is particularly the case for South Africa (SA) with its very high level of income inequality and inequalities in health and health outcomes. This paper provides evidence on the key SDH for reducing health inequalities in the country using a framework initially developed by the World Health Organization.

**Objective:**

This paper assesses health inequalities in SA and explains the factors (i.e. SDH and other individual level factors) that account for large disparities in health. The relative contribution of different SDH to health inequality is also assessed.

**Design:**

A cross-sectional design is used. Data come from the third wave of the nationally representative National Income Dynamics Study. A subsample of adults (18 years and older) is used. The main variable of interest is dichotomised good versus bad self-assessed health (SAH). Income-related health inequality is assessed using the standard concentration index (CI). A positive CI means that the rich report better health than the poor. A negative value signifies the opposite. The paper also decomposes the CI to assess its contributing factors.

**Results:**

Good SAH is significantly concentrated among the rich rather than the poor (CI=0.008; *p*<0.01). Decomposition of this result shows that social protection and employment (contribution=0.012; *p*<0.01), knowledge and education (0.005; *p*<0.01), and housing and infrastructure (−0.003; *p*<0.01) contribute significantly to the disparities in good SAH in SA. After accounting for these other variables, the contribution of income and poverty is negligible.

**Conclusions:**

Addressing health inequalities *inter alia* requires an increased government commitment in terms of budgetary allocations to key sectors (i.e. employment, social protection, education, housing, and other appropriate infrastructure). Attention should also be paid to equity in benefits from government expenditure. In addition, the health sector needs to play its role in providing a broad range of health services to reduce the burden of disease.

Globally, it is recognised that health and health outcomes are not only affected by healthcare or access to health services. They result from multidimensional and complex factors linked to the social determinants of health (SDH) (1–3), which include a range of social, political, economic, environmental, and cultural factors, including human rights and gender equality (1). Although ‘health may not be the main aim of policies in these sectors, they have strong bearing on health and health equity’ including inequalities in health (4). Thus, tackling health inequalities will require action on the SDH and an understanding of the pathways by which SDH influence population health within a specific country context (3, 5–10). However, evidence of the impact of inter-sectoral action (i.e. including several sectors in addition to the health sector when designing and implementing public policies to improve quality of life) (11) on health inequality and (in)equity^1^ is limited and there is a call for rigorous evaluations of inter-sectoral action to strengthen the evidence base (15). This call is of great relevance for Africa given its huge burden of disease.

In the case of South Africa (SA), there exist wide inequalities in the distribution of health and health outcomes (16–19). This is also the case with the SDH. For instance, income inequality in the country is among the highest in the world (20) and high levels of poverty and unemployment (21, 22) exist. SA's gender inequality index was estimated at 0.461 (2013), and it is ranked 94th out of the 152 countries with data (23). These inequalities, to some extent, could be historically linked to the colonial and apartheid legacy (19, 24).

Furthermore, SA faces a quadruple burden of disease (tuberculosis, HIV, and AIDS; high levels of maternal and child mortality and other communicable diseases; injuries; and non-communicable diseases, or NCDs) (25) that is linked to poverty and deprivation. In 2011, NCDs alone accounted for 34.4% of years of life lost (26).

It is only recently that there has been a greater recognition of the role of inter-sectoral action in tackling some of the health challenges facing SA (27). For example, the Negotiated Service Delivery Agreement between the Minister of Health and the President (28) recognises the need for addressing the SDH (29). In addition, a key emphasis in the first phase of the National Health Insurance (NHI) reforms in SA is on ensuring that not only curative, but also preventive and promotive primary health services, which engage other relevant sectors, are dramatically improved. However, there is little in the way of a clear strategy that addresses health inequalities bearing in mind the contributions of different sectors to health inequality in the country (19). This puts SA in a situation where there is a need for evidence to inform strategies for collaborative inter-sectoral action to redress existing health inequalities.

In order to inform policy on key SDH for reducing health inequalities, this paper seeks to assess health inequalities in SA and to explain the factors (i.e. the SDH) beyond healthcare supply-side features that account for large disparities in health in the country. Specifically, the paper assesses the relative contribution of different SDH to health inequality in SA. It uses domains and subdomains that were originally identified as part of a collaborative project with the World Health Organization (WHO) to monitor inter-sectoral factors that influence equity-oriented progress towards Universal Health Coverage and health equity. The domains include income and poverty, knowledge and education, housing and infrastructure, travel, community and infrastructure, social protection and employment, early childhood development, gender norms, autonomy and participation, registration, accountability, and discrimination (Table 1). The original domains have been consistently refined and condensed into three broad coherent domains – environmental quality, accountability and inclusion, and livelihoods and skills (30).^2^ The analyses in this paper use a framework based on the original domains, which remain compatible with the new groupings.

**Table 1:** Summary description of variables

Domains	Description	Mean/percentage
**Income and poverty**		
Per capita household income	Average annual household per capita income. Household per capita income proxied by household expenditure	$3,000.00
Income	Ratio of average household per capita income to household reported per capita income^a^	1.53
Poor household with at least one child	A household living below the poverty line (R466/month)^b^ with at least one child less than 5 years (1=poor with at least one child; 0=otherwise)	6.25%
Poor household with at least one adult above retirement age	A household living below the poverty line (R466/month)^b^ with at least one adult aged 65+ years (1=poor with at least one adult above retirement age, 0=otherwise)	2.41%
**Knowledge and education**		
Adult completing at least secondary education	An adult (18+ years) that has completed at least secondary education (1=completed secondary education; 0=otherwise)	36.83%
**Housing and infrastructure**		
Composite housing	Collection of five variables at the household level:	
	(1) good sanitation (1=good sanitation; 0=otherwise)	78.46%
	(2) electricity available in household (1=electricity available; 0=otherwise)	87.32%
	(3) clean cooking source (1=clean source; 0=otherwise)	84.65%
	(4) clean lighting source (1=clean source; 0=otherwise)	89.76%
	(5) clean drinking water source (1=clean source; 0=otherwise)	93.09%
**Community and infrastructure**		
Community infrastructure	A collection of two variables:	
	(1) having street lighting around the household where the individual lives (1=street lighting available; 0=otherwise)	54.45%
	(2) regular rubbish/refuse removal by local authorities (1=regular removal; 0=otherwise)	65.78%
Safety	A binary variable denoting the absence or very infrequent occurrence of all of the following within the neighbourhood of the household: theft/burglary; violence among households; gangsterism, murder, shooting and stabbing in neighbourhood; and drug abuse in neighbourhood (1=safe neighbourhood; 0=otherwise)	14.73%
**Social protection and employment**		
Private health insurance	Private health insurance membership (1=member; 0=non-member)	15.63%
Grant recipient	Receiving any form of social assistance from the government (1=receives social assistance; 0=otherwise)	52.03%
Employed adult	Being in paid employment: i.e. self-employed or working for pay as an employee or employer (1=employed; 0=not employed)	40.79%
**Gender norms**		
Female DM1	From a household where a female member is principally responsible for making decisions about day-to-day household expenditures (1=female decision maker household; 0=otherwise)	56.12%
Female DM2	From a household where a female member is principally responsible for making decisions about large, unusual purchases (1=female decision maker household; 0=otherwise)	48.03%
Female DM3	From a household where a female member is principally responsible for making decisions about where children should go to school (1=female decision maker household; 0=otherwise)	40.49%
Female-headed household	Household is headed by a female (female household head=1; male household head=0)	54.17%
**Other individual/household-specific factors**		
Race	Race category:	
	(1) White	9.67%
	(2) Black	78.69%
	(3) Coloured	9.16%
	(4) Indian/Asian	2.49%
Provincial dummies	Province of residence of the individual (i.e. 9 dummy indicators corresponding to the 9 provinces in SA)	–
Rural	Location of individual (1=rural area; 0=urban area)	34.49%
Household size	The number of individuals living in the household. Household membership is defined as ‘spending more than 15 days in the last 12 months at the household and sharing food and resources when staying at that household’ (32)	3.27
Age	Age in years	40.08
Female	Sex (1=female; 0=male)	54.14%
Illness	Self-reported illness or injury in the past 30 days (1=reported illness/injury; 0=otherwise)	57.19%
Good SAH	Self-assessed health status (1=reporting good, very good or excellent; 0=fair or poor)	88.16%

Some of the original domains are not included. Registration domain is omitted due to redundancy as data indicate that the coverage of birth registration is in excess of 98%. Early childhood development domain is not contained here because only data on adults were used. The travel, participation, accountability, and discrimination domains are not contained here because data or proxies are not available. However, some of the variables such as race, location, sex, and other socio-economic status variables pick up some information on discrimination.

a This procedure was adopted to ensure that the indicators for income and poverty follow the same direction. A ratio greater than one signifies households with income less than the average income.

b The average nominal exchange rate is $1=R10. Although SA does not have a unique national poverty line, one of the widely used poverty lines (47) was updated and used to categorise households as poor/non-poor.

## Methodology

### Data

Data come from the third wave of the nationally representative National Income Dynamics Study (NIDS) (31). The NIDS is a longitudinal survey that uses a two-stage sampling design. In the first stage, a sample of about 400 primary sampling units (PSUs) is drawn from a Statistics South Africa master sample of about 3,000 PSUs. The PSUs are non-overlapping and they comprise the first sampling units from which households or dwellings are selected. In the second stage, dwellings within each PSU are selected. The third wave (2012/13) contains information on a total of about 10,000 households and data were collected between April and December 2012. The NIDS is designed to assess and track the dimensions of well-being over time and thus provides a rich and reliable source of data on variables related to the proposed framework (Table 1). The NIDS panel data suffer from attrition which is due mainly to household non-response. In the third wave, the attrition rate was about 15.9% (32). Although this may have an effect on the estimates, the sampling weights have been adjusted to account for attrition (32).

The survey covers private households in the country (including residents in workers’ hostels, convents, and monasteries). Trained enumerators were used to collect the data using three sets of questionnaires at both the individual and household levels. One set of questionnaires was designed for adults (people aged 15 years and older) and completed by the individual where possible; another set was designed for children younger than 15 years and was completed by the mother or caregiver of the child; the third set was a household questionnaire completed by the oldest woman in the household. Data collected include basic demographic information on all household members, education, labour market participation, health (including anthropometric measures), household decision making, well-being and social cohesion, and household expenditure and income.

This paper uses a sample of adults (18 years and older) in the analysis (i.e. about 16,000 adults). This is because secondary school completion, an important variable in the analysis, is only expected after age of 18 years.

### Analytical method

Self-assessed health status (SAH) is used as a measure of health. In the NIDS, respondents rated their health status in one of the following five categories: excellent, very good, good, fair, or poor.^3^ This was further dichotomised to obtain ‘good health’ (i.e. corresponding to excellent, very good, and good). Although debatable, literature has noted that ‘income-related inequality in SAH is unlikely to be biased by reporting error’ and ‘inequalities in SAH by income do have predictive power for inequalities in survival by income’ (33, p. 1621). SAH is multidimensional as it implicitly includes *inter alia* physical, functional, coping, and well-being aspects (34). This further serves as a motivation for its choice over individual disease conditions such as diabetes, tuberculosis, and injuries or other self-reported illnesses. Moreover, SAH is validated as a predictor of mortality and morbidity (35, 36).

Income-related inequality in health is assessed using the concentration index (*CI*_*H*_). Generally, many researchers analyse inequality (absolute and relative) using six widely available measures (i.e. the range, the Gini coefficient, a pseudo-Gini coefficient, the index of dissimilarity, the slope index of inequality, and the CI) (37, 38). To assess relative inequality using a bivariate analysis involving a measure of income, only the slope index of inequality and the CI are consistent with ranking units across socio-economic groupings. They are also sensitive to changes in population distribution across socio-economic groups and consistent with experience of health (or ill health) across the distribution of socio-economic groups (37, 38).

For empirical estimation, the *CI*_*H*_ is computed simply as twice the covariance between the health variable and the individual's relative rank based on per capita income, divided by the mean of the health variable (38). The paper uses the Distributive Analysis Stata Package routine (39) to estimate the concentration indices in Stata^®^ (40). In theory, the index lies between −1 (i.e. a case where all the population's ‘good health’ is concentrated on the most disadvantaged individual) and +1 (when it is concentrated on the least disadvantaged individual) (37). In general, a positive index corresponds to a pro-rich distribution of ‘good health’ whereas a negative index corresponds to a pro-poor distribution of ‘good health’. Household per capita expenditure is used as a proxy for relative income.

In order to assess the relative contributions of the proposed domains to health inequality, the *CI*_*H*_ is further decomposed using the Wagstaff, van Doorslaer (41) methodology. Here, *CI*_*H*_ can be decomposed and written equivalently as follows:

1 $$CI_{H}=\Sigma_{y}\underset{\text{Deterministic}}{\underset{︸}{C_{y}}}+\underset{\text{Unexplained}}{\underset{︸}{\varepsilon }}$$

where $C_{y}=CI_{y}\left(\frac{\beta_{y}{\bar{z}}_{y}}{\mu_{H}}\right)$ and $\varepsilon =\left(\frac{GC\varepsilon }{\mu_{H}}\right)$. The residual, *ɛ* should approach zero in a well-specified model. The CI and mean of the factor or domain *y* are *CI*_*y*_ and ${\bar{z}}_{y}$, respectively. The mean of good SAH is given as *µ*_*H*_. The generalised CI of the error term (*ɛ*) is *GC*_*ɛ*_ and *β*_*y*_ is the coefficient of factor *y* obtained from a linearly additive equation that relates the contributing factors (*y*), shown in Table 2, to good SAH (*H*). That is:

2 $$H=\alpha +\sum \beta_{y}y+\varepsilon$$

**Table 2:** Decomposition of health inequality, South Africa, 2012/13

	Concentration index^a^ (*a*)	Elasticity (*b*)	Contribution^b^ (*c*)=(*a*)×(*b*)
**Income and poverty**			
Income	−0.5318*** (0.0047)	−0.0009	0.0005 (0.0018)
Poor household with at least one adult above retirement age	−0.6322*** (0.0122)	0.0012	−0.0007* (0.0004)
Poor household with at least one child	−0.6749*** (0.0091)	−0.0002	0.0001 (0.0006)
**Knowledge and education**			
Adult completing at least secondary education	0.3026*** (0.0094)	0.0161	0.0049*** (0.0011)
**Housing and infrastructure**			
Good sanitation	0.1093*** (0.0033)	−0.0054	−0.0006 (0.0008)
Electricity available in household	0.0432*** (0.0024)	−0.0077	−0.0003 (0.0007)
Clean cooking source	0.0828*** (0.0026)	0.0004	0.00003 (0.0010)
Clean lighting source	0.0420*** (0.0020)	−0.0367	−0.0015* (0.0008)
Clean drinking water source	0.0316*** (0.0016)	−0.0210	−0.0007** (0.0003)
**Community and infrastructure**			
Having street lighting where the individual lives	0.2065*** (0.0068)	−0.0069	−0.0014 (0.0014)
Regular rubbish/refuse removal by local authorities	0.1894*** (0.0050)	0.0153	0.0029* (0.0016)
Safety	−0.1052*** (0.0179)	0.0010	−0.0001 (0.0002)
**Social protection and employment**			
Private insurance	0.6362*** (0.0126)	0.0034	0.0022 (0.0015)
Grant recipient	−0.2812*** (0.0088)	−0.0125	0.0035*** (0.0014)
Employed adult	0.2621*** (0.0087)	0.0233	0.0061*** (0.0012)
Gender norms			
Female DM1	−0.0939*** (0.0080)	−0.0107	0.0010 (0.0008)
Female DM2	−0.1304*** (0.0093)	−0.0014	0.0002 (0.0009)
Female DM3	−0.1958*** (0.0098)	−0.0036	0.0007 (0.0010)
Female-headed household	−0.1181*** (0.0085)	0.0059	−0.0007 (0.0007)
**Other individual/household-specific factors**			
Age	0.0199*** (0.0034)	−0.1555	−0.0031 (0.0021)
Age squared	0.0286*** (0.0068)	−0.0476	−0.0014 (0.0017)
Female	−0.0763*** (0.0090)	−0.0061	0.0005 (0.0003)
Household size	−0.2338*** (0.0046)	0.0205	−0.0048*** (0.0016)
Illness	0.0056 (0.0081)	−0.0788	−0.0004 (0.0005)
Rural	−0.3080*** (0.0106)	−0.0017	0.0005 (0.0014)
Race	–	–	0.0003 (0.0002)
Provincial dummies	–	–	0.0003 (0.0002)
Residual			0.0001 (0.0027)
Concentration index			0.0080*** (0.0030)

a Analytical standard errors are in parenthesis;

b bootstrap standard errors in parenthesis using 1,000 replications;

* *p*<0.10,

** *p*<0.05,

*** *p*<0.01.

In summary, the methodology decomposes inequality by estimating the contribution of each underlying factor or domain *y* (*C*_*y*_) to health inequality (*CI*_*H*_). Using a regression model [see Equation (2)] that links SAH to a set of health determinants and domains (*y*), the contribution of a factor (e.g. education) (*C*_*y*_) to income-related health inequality is the product of the degree of responsiveness of health to changes in the factor^4^ $\left(\frac{\beta_{y}{\bar{z}}_{y}}{\mu_{H}}\right)$ and the degree of income-related inequality in that factor (*CI*_*y*_) measured using the CI.

Generally, when the contribution of factor *y* to disparities in good SAH is positive (i.e. *C*_*y*_>0) and the contribution of factor *y* is *x* % to inequality in health, it means that, all things being equal, income-related disparities in good SAH would be *x* % lower if factor *y* is either equally distributed across the income range or if the elasticity is zero (i.e. *E*_*y*_=0) (42). The reverse is the case when the contribution is negative (i.e. *C*_*y*_<0).

Table 1 contains a list of the factors included in our analysis, based on the literature on determinants of health (6, 8, 42–44), and the original 12 domains. These factors, which are generally regarded as key SDH, include those that relate to the different sectors which are likely to affect health. They are used to assess the relative contributions of different factors and sectors to health inequalities in SA.

Standard errors of the contribution of the various factors to the CI are obtained using bootstrap methods with 1,000 replications taking into account the sampling structure of the data set (45, 46). This is because analytical expressions cannot be written for the standard errors. All statistical analyses were conducted using Stata^®^ version 13 (40) using poststratification weights to account for attrition in the NIDS data set (32).

## Results

As shown in Table 1, more than 54% of the adult population are women and the average age of the adults is slightly above 40 years. The majority of the population (>78%) is of black/African descent, whereas the Indian/Asian population is the smallest (<3%). Two-third of the population is located in urban areas. Slightly more than 57% of the adult population reported an illness or injury in the 30 days preceding the survey while over 88% assessed their health status to be at least good. On average, only 6.2% of households are poor with at least one child aged less than 5 years whereas only 2.4% of households are poor with at least one adult aged 65 years and older. Educational attainment is very low among adults as only about 37% have completed secondary education. Access to good sanitation, electricity, clean source of drinking water, and clean cooking source is high (>78%) among households. However, community infrastructure such as streetlights (only 54% of households) and regular refuse removal by local authorities (66% of households) are not widely reported. Safety is a major concern within neighbourhoods, with less than one-sixth of households living in a safe neighbourhood. Although only about 41% of the adults are employed (Table 1), more than half receive government social assistance in the form of grants. These grants include child support grants, old age pensions, disability grants, foster care grants, and care dependency grants. Only one-sixth of the adult population has private health insurance membership.

Proxies of female empowerment vary across indicators. Although a female is the household head in about 54% of households, only 40% of households allow women to be principally responsible for decisions about the school that children will attend. However, females are principally responsible for making decisions about day-to-day household expenditures in about 56% of households, which is greater than their share in household headship. On the other hand, less than 50% of households allow women to be principally responsible for making large and unusual household expenditures (Table 1). Interestingly, as shown in Table 2, female-headed households are significantly concentrated in poorer households (CI=−0.118; *p*<0.01). Households where females are principally responsible for decisions about day-to-day purchases (CI=−0.094; *p*<0.01), choice of school for children (CI=−0.196; *p*<0.01), and unusual large expenses (CI=−0.130; *p*<0.01) are also significantly concentrated among poorer households.

The receipt of government grants (CI=−0.281; *p*<0.01), larger household size (CI=−0.234; *p*<0.01), and the prevalence of ‘safe’ neighbourhoods (CI=−0.105; *p*<0.01) are significantly concentrated among the poor. On the other hand, completion of secondary education (CI=0.303; *p*<0.01), being employed (CI=0.262; *p*<0.01), purchase of private health insurance coverage (CI=0.636; *p*<0.01), and other housing and communal amenities are generally significantly concentrated among the rich (Table 2).

**Fig. 1:**
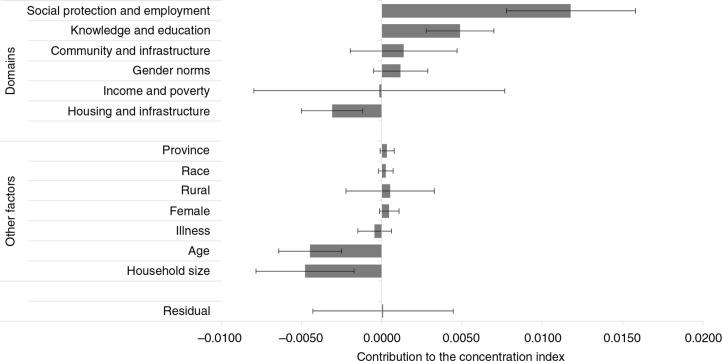
The contribution of social determinants of health to health inequality in South Africa, 2012/13. The error bars represent 95% confidence intervals based on bootstrapped standard errors using 1,000 replications.

The CI of good SAH (CI=0.008; *p*<0.01) indicates that good health is significantly concentrated among the rich rather than among the poor (Table 2). The gradient implies that, on average, poorer adults report poorer states of health compared to richer adults. A decomposition of this result (Table 2) shows that significant factors contributing to the disparities in good SAH include employment status (contribution=0.006; *p*<0.01), receiving government grants (0.004; *p*<0.01), household size (−0.005; *p*<0.01), completing secondary education (0.005; *p*<0.01), access to clean drinking water (−0.001; *p*<0.05), clean source of lighting (−0.002; *p*<0.10), poor household with a child (−0.001; *p*<0.10), and regular rubbish/refuse removal by local authorities (0.003; *p*<0.10). Factors such as income, age, sex, rural location, and race do not make significant contributions to the disparities in good SAH. Such lack of a significant contribution may arise in some cases where the effect of some of the variables (e.g. income) on health inequality has been captured through the other significant variables (e.g. employment status). However, as shown in Fig. 1, the aggregation of some of these factors under the broad domains may result in their significant contribution to disparities in good SAH.

Using the broad domains contained in Table 1, and as shown in Fig. 1, the major significant domains that contribute to disparities in good SAH in SA include social protection and employment (contribution=0.012; *p*<0.01), knowledge and education (0.005, *p*<0.01), and housing and infrastructure (−0.003; *p*<0.01). The social protection and employment domain alone contributes over 149% to the concentration of good SAH among rich adults in SA (Table 3). Knowledge and education, and housing and infrastructure contribute about 62% and −39% to disparities in good SAH, respectively (Table 3). The contributions of the other domains where data are available are not statistically significant at conventional levels. Surprisingly, the contribution of the income and poverty domain is negligible in this analysis. However, many of the other domains are related to income (such as employment and receipt of grants) and poverty (such as lack of clean water, good housing, and education).

**Table 3:** Percentage contribution of the ‘domains’ to health inequality in South Africa, 2012/13

	Percentage contribution to the concentration index
Social protection and employment	149.33
Knowledge and education	61.78
Community and infrastructure	17.27
Gender norms	14.96
Income and poverty	−1.90
Housing and infrastructure	−39.23

## Discussion

The life course approach is helpful in understanding health inequities and health inequality as these may vary with age (44). This paper has shown, using an adult population (18 years and older), that inequalities in good health are to the advantage of the rich in SA. This has traditionally been understood as the health gradient (16, 48–50) in the literature. Within the framework of the initial domains proposed by the WHO including their further refinements (30), three major significant domains or areas of SDH action emerged from the analysis in SA. These domains (knowledge and education, social protection and employment, and housing and infrastructure) account for significant disparities in good health in the country.

In a resource book that aims to ‘guide policymakers on how to present the case for action on the SDH and the social determinants of unfair, avoidable health inequalities (health inequities)’ the WHO highlights three sectors (i.e. education, social protection, and urban development and infrastructure) as critical for action on the SDH (44). These sectors were identified *inter alia* for the potential impacts that inter-sectoral action will have on health outcomes and health inequalities; empirical evidence on what interventions and sectors have an impact on health; and the likelihood of government action through the different government levels (44). The results of this paper clearly highlight these same three sectors as key domains for inter-sectoral action to reduce health inequalities, at least among the adult population in SA.

Policy action in these areas requires substantial government involvement and funding. An appropriate policy response to inequalities in health and SDH is to prioritise improvements in access to the full range of social services and basic amenities identified in this paper.

**Fig. 2:**
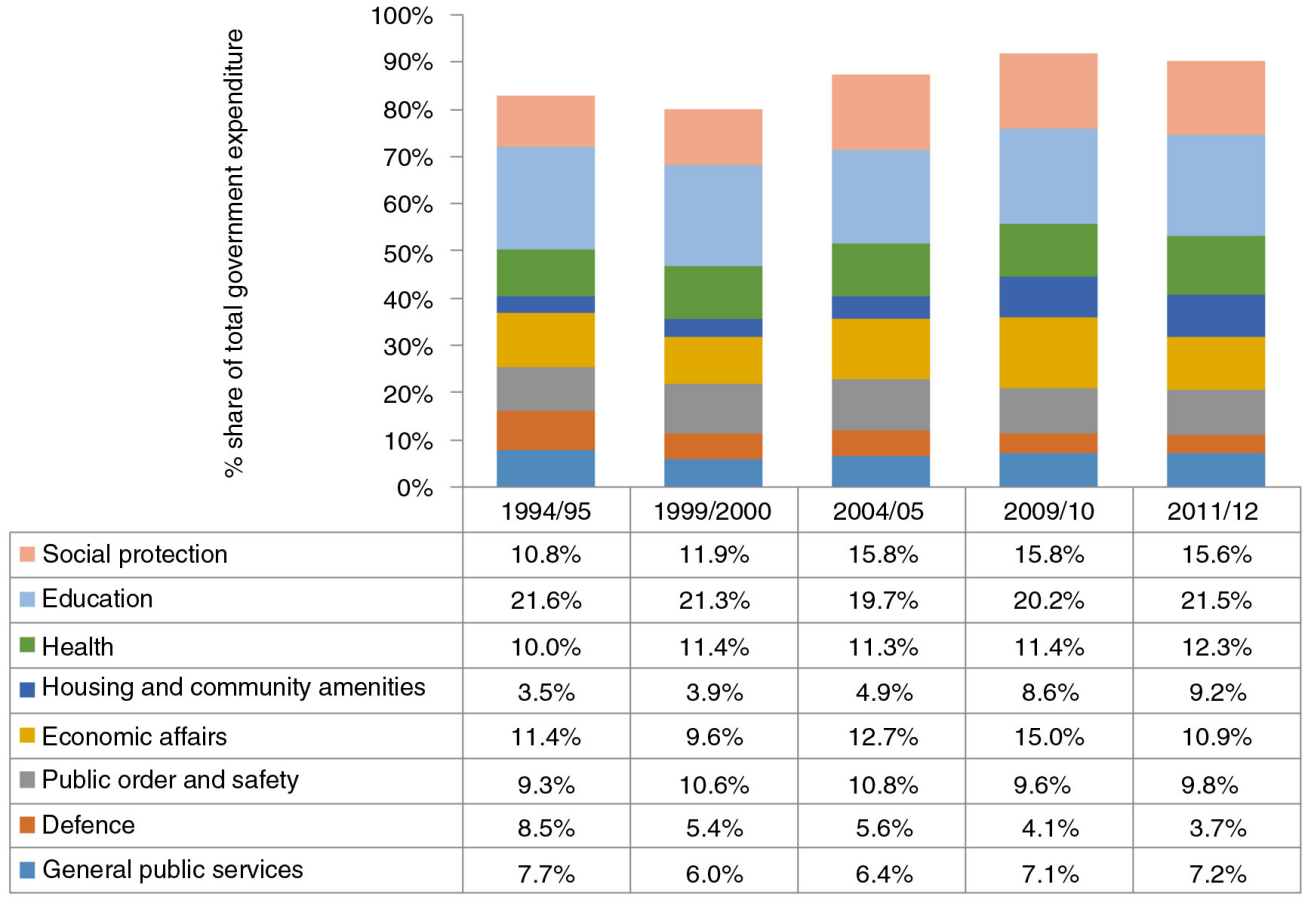
Selected public expenditure categories as a share of total public expenditure, South Africa 1994–2012. From ref. (51).

Allocations to these ‘SDH-priority’ government sectors in 2010–2011 (Fig. 2) indicate that social protection (15.6%), education (21.5%), and housing/community amenities (9.2%) account for nearly half of government expenditure (51). Although there is evidence of the positive impact of the social protection policy in the form of government social assistance in the country (52, 53), it is unclear whether public expenditure on education and housing/community amenities has translated into substantial improvements for the worst-off since 1994. There are considerable disparities across the country in the efficiency and quality of service delivery provided from the same resources per capita (26). It is the case that poorer South African students perform worse academically (54) and poorer households have poorer housing amenities (55), and as the results of this paper show, secondary school completion is significantly concentrated among richer South Africans.

Given the magnitude of inequalities in all aspects of life in SA inherited from the colonial and apartheid eras (24), fiscal policy decisions are critical. This relates not only to the distribution of available government resources across sectors but also to the overall level of government expenditure. In 1996, the South African government adopted the growth, employment, and redistribution policy (56), in which it committed itself to not increasing the tax to gross domestic product (GDP) ratio. Since then, tax revenue has been maintained at around 25% of GDP consistently, despite substantial improvements in tax collection efficiency and tax compliance over this period (57). Maintaining the 25% tax to GDP ratio has been achieved by reducing personal and corporate tax rates (e.g. corporate tax rates have been reduced from 35% in the mid-1990s to 28% at present) (57). Given that the budget deficit has also been dramatically reduced over this period, there have been severe constraints on real government expenditure levels, particularly between the mid-1990s and mid-2000s (24). In order to make more rapid progress on addressing health and other inequalities and the SDH in SA, a critical review of the fiscal policy may be required.

Because employment is a key determinant of health, there is also a need for the country to create employment to absorb the large number of unemployed youth. With a high unemployment rate and a relatively low prevalence of informal employment (58), the case of SA is a dilemma. The unemployed are not absorbed into informal employment because of some rigidities relating to national and local regulations (58, 59). To generate employment in SA, among other things, the economy needs to grow. Evidence from the period immediately after the financial crisis (2008–2012) (i.e. a period that witnessed a decline in economic growth), shows that a 10% increase in GDP led to a 1.6% reduction in employment. But between 2000 and 2008, a 1% increase in GDP produced a 0.69% increase in employment in SA (59). Apart from growing the economy, it is important to recognise that unemployment in SA is mainly structural, meaning that it is very difficult, in the short term, to eliminate it without ensuring substantial investments in education and training. This links back to the issue of education emphasised earlier.

Apart from fiscal policy issues and inter-sectoral actions, there is a need within the health sector to also pay attention to health promotion and prevention. Part of this realisation is contained in the South African government's commitment to moving to a universal health system, through introducing an NHI Fund (60). The NHI system is intended to be tax funded with an NHI Fund proposed for strategic purchasing of health services for the entire population. Although the proposed changes in healthcare financing arrangements will take some time to implement, the first phase of reforms is already well underway. This entails considerable investment in improving the accessibility and quality of services at the primary care level. Within this first phase also, preventive and promotive primary care services are to be dramatically improved with a focus on inter-sectoral action within the SDH framework.

One of the major strengths of the paper is the focus on general health status, rather than focusing on morbidity from individual diseases. However, this is a self-assessed measure and it may suffer from bias that may be systematically related to income or other measures of socio-economic status. Nonetheless, the results of the paper are in line with those in the literature regarding the so-called ‘health gradient’. There is also a loss of information in the dichotomisation of the response categories which may affect the inequality estimated. Although this is difficult to assess, the selected dichotomisation is the most reasonable one given the nature of the original five categories. The excellent, very good, and good categories have been recoded as good SAH. Also, interaction terms could be used to account for differential effects that a factor may have on good SAH depending on the values of another factor when estimating equation (2) (61). However, it will be difficult to retrieve the ‘pure’ slope coefficients for each factor as this will depend on the values of another variable (i.e. the interacting variable). In general, in regression analysis, some researchers may use an estimate of any of the measures of central tendency (e.g. the mean) to compute the slope. Clearly, the choice of any value has an impact on the estimated slope.

A multilevel analysis with the inclusion of social capital indicators should ideally be used to examine the role of the SDH on health inequality. However, previous research using a multilevel analytical framework and the NIDS data set has shown that ‘the bulk of the variations in the data occur at the individual level rather than at the higher levels’ (43) thus not requiring the imposition of a hierarchical structure. In that paper, the only significant social capital variables that explained SAH were a community service group membership and personalised trust.

## Conclusions

The results of this paper have shown that although the disparities in good health are to the advantage of the rich, sectors other than health have a substantial and significant impact on health inequalities in SA. Social service sectors and domains including social protection and employment, education, and housing and community amenities contribute significantly to inequality in good health in SA. Thus, policy interventions in these areas are not only likely to lead to improvements in their own domains alone but to health as well. Within the context of SA, the paper argues for an increase in government's role within these areas in order to redress historic health inequalities and inequities in the country. Thus, government must target expenditures at the disadvantaged and it must also ensure an equitable distribution of expected outcomes. The health sector has an important role to play within the proposed NHI Fund, not only through ensuring that all have improved access to quality health services but also that preventive and promotive services receive attention in order to reduce disease burden. If adequate attention is paid to these areas within the broad framework of the SDH, SA is likely to make substantial advancements in reducing health inequalities and the country will be put on the path to sustainable development.

## Footnotes

1 Health inequality (i.e. disparities in health between population groups) as used in this paper has a bivariate connotation in such a way that investigates whether people with ‘better’ health also have high income or material well-being (12). On the other hand, health inequities refer to such disparities that are unnecessary, avoidable, unfair, and unjust (13). Thus, health inequities ‘systematically put groups of people who are already socially disadvantaged … at further disadvantage with respect to their health’ (14, p. 254).

## Footnotes

2 Because this paper is part of a series of papers that focus on applying the same framework, readers are referred to the other papers in the series that provide a general overview of the processes involved in identifying the domains and how the current categorisation of domains has evolved.

## Footnotes

3 In the NIDS, SAH was elicited using the question ‘How would you describe your health at present? Would you say it is excellent, very good, good, fair, or poor?’

## Footnotes

4 Economists refer to the degree of responsiveness of a factor to changes in another factor as elasticity.
